# Clinical therapeutic effects of AO/ASIF clavicle hook plate on distal clavicle fractures and acromioclavicular joint dislocations

**DOI:** 10.12669/pjms.304.5269

**Published:** 2014

**Authors:** Qingjun Dou, Xiaofeng Ren

**Affiliations:** 1 Qingjun Dou, Department of Orthopedics, Liaocheng People's Hospital and Liaocheng Clinical School of Taishan Medical University, Liaocheng 252000, PR China.; 2 Xiaofeng Ren, Department of Orthopedics, Liaocheng People's Hospital and Liaocheng Clinical School of Taishan Medical University, Liaocheng 252000, PR China.

**Keywords:** AO/ASIF claviclehook plate, Distal clavicle fracture, Acromioclavicular joint dislocation, Therapeutic effect

## Abstract

***Objective:*** The aim of this study was to evaluate the security and effectiveness of AO/ASIF clavicle hook plate in the treatment of distal clavicle fractures and acromioclavicular joint dislocations.

***Methods:*** One hundred patients with distal clavicle fractures and acromioclavicular joint dislocations who were admitted in our hospital from January 2012 to January 2013 were selected as the study subjects. They were then randomly divided into a control group and an observation group (n=50). The observation group was treated with AO/ASIF clavicle hook plates, and the control group was treated with Kirschner-wire tension bands. The outcomes were recorded and compared.

***Results:*** The JOA scores of the two groups were similar before surgery (P>0.05). The two groups both had obviously increased JOA scores in the postoperative 6th and 12th weeks, and the score in the postoperative 12th week was higher. There were statistically significant intra-group differences (P<0.05). The postoperative 6th-week and 12th-week JOA scores of the observation group were (83.2±1.8) and (97.4±1.5) respectively, and those of the control group were (71.6±2.2) and (82.3±2.6) respectively, with statistically significant inter-group differences (P<0.05). Significantly more patients in the observation group (100%) were evaluated as excellent or good outcomes after fixation than those in the control group (60%). After removal of the surgical apparatus, the recurrence rates of bone fracture and joint dislocation in the observation group were significantly lower than those of the control group (P<0.05).

***Conclusion:*** AO/ASIF clavicle hook plate functioned more effectively than Kirschner-wire tension band in clinical treatment of distal clavicle fractures and acromioclavicular joint dislocations. The former protocol enjoyed small incisions, firm fixation and early shoulder mobility. Therefore, it is a safe and effective surgical method that is worthy of being widely applied in clinical practice.

## INTRODUCTION

Distal clavicle fractures and acromioclavicular joint dislocations, as common shoulder injuries in clinical practice, are mostly induced by violence. The patients with bone fracture and dislocation are often complicated with injuries in acromioclavicular ligament and coracoclavicular ligament.^1^ Shoulder injuries are generally subjected to conservative treatment and surgical treatment. Although ensuring satisfactory recovery and aesthetically pleasing appearance, the former treatment does not function well for the patients with distal clavicle fractures and acromioclavicular joint dislocations. Nowadays, such patients are mainly treated surgically with Kirschner-wire tension band, T-type anatomical plate and miniature external fixator. However, these methods are prone to inducing complications.^2^

In contrast, AO/ASIF clavicle hook plate has become a promising protocol for treating the patients mentioned above. Therefore, the study aimed to evaluate the therapeutic effects of AO/ASIF clavicle hook plate on the patients with distal clavicle fractures and acromioclavicular joint dislocations.

## METHODS

***Clinical data: ***One hundred patients with distal clavicle fractures and acromioclavicular joint dislocations who were admitted in our hospital from January 2012 to January 2013 were selected as the study subjects. They were then divided into a control group and an observation group (n=50) by random number table method. The observation group was treated with AO/ASIF clavicle hook plates, and the control group was treated with Kirschner-wire tension bands. The observation group comprised 38 males and 12 females who were aged 25-52 years old [average: (37.5±3.3)]. Bone fracture types: 33 cases of distal clavicle fractures (Tossy type III) and 17 cases of acromioclavicular joint dislocations (Neer type II). The control group comprised 35 males and 15 females who were aged 24-53 years old [average: (36.3±3.7)]. Bone fracture types: 31 cases of distal clavicle fractures (Toss type III) and 19 cases of acromioclavicular joint dislocations (Neer type II). The two groups were treated within 3 h-7 d after injuries. The gender, age and bone fracture types of the two groups did not differ significantly (P>0.05). This study was approved by the ethics committee of our hospital, and written consent has been obtained from all patients.

***Methods: ***The observation group was treated with AO/ASIF clavicle hook plates following the procedure below. After cervical plexus anesthesia, the patient's head was moved towards the healthy side while raising the injured side in his/her supine position, and then an approximately 10 cm-length curved incision was made along the clavicle from the acromion to completely expose the acromioclavicular joint and the distal clavicle fracture. First, hematomas and fracture fragments were eliminated, and acromioclavicular joint was relocated by outreaching and raising the shoulder. Thereafter the clavicle hook plate was shaped to be adaptable to the clavicle, and the hook end was fixed under the acromion while keeping tight fitting between the steel plate and the distal clavicle. Finally, the steel plate was fixed on the clavicle with 3.5 mm titanium screws after drill punching. Subsequently, the injured ligament was repaired, and the incision was closed layer-by-layer after place drains. The control group was subjected to Kirschner-wire fixation. The preoperative preparation, anesthesia method and incision were the same as those of the observation group. Meanwhile, hematomas and fracture fragments should also be eliminated first, and the joint was fixed by a forceps holder after relocation. A total of two Kirschner wires were introduced in an antegrade direction along the lateral clavicle between the acromion and the clavicle. After the wires were bent and buried under skin, the wound was rinsed and sutured layer-by-layer. After four weeks of suspension, the shoulder joint was moved properly under doctor's instructions. The two groups were followed up for 3-24 months after surgery.

***Observation indices: ***Therapeutic effects were determined according to the Karlsson standard.^3^ Excellent: Painless in the shoulder joint, recovered shoulder activity and upper limb muscle strength, and 4 mm acromioclavicular joint space disclosed by X-ray examination. Good: Painless or mild pain in the shoulder joint, partially recovered shoulder joint function, mildly limited activity, Grade 4 upper limb muscle strength, and 7 mm acromioclavicular joint space disclosed by X-ray examination. Poor: Intense pain or pain at night in the shoulder joint, obviously limited shoulder joint activity, Grade 3 upper limb muscle strength, and 8 mm acromioclavicular joint space disclosed by X-ray examination. The shoulder joint functions of the two groups were compared by JOA scores before surgery as well as in the postoperative 6th and 12th weeks.^4^ In addition, after removal of the fixing apparatus, the recurrence rates of bone fracture and joint dislocation were recorded and compared.

***Statistical analysis: ***All data were analyzed by SPSS 15.0. The numerical data were compared by χ^2^ test, and the categorical data were compared by t test. P<0.05 was considered statistically significant.

## RESULTS

***JOA scores before and after surgery: ***The JOA scores of the two groups were not significantly different before surgery (P>0.05). However, the scores were remarkably elevated in the postoperative 6th and 12th weeks, and the score in the postoperative 12th week was higher. There were statistically significant intra-group differences (P<0.05). The postoperative 6th-week and 12th-week JOA scores of the observation group were significantly higher than those of the control group at each time point, with statistically significant inter-group differences (P<0.05) (Table-I).

***Evaluation on therapeutic effects after surgery: ***The observation group had significantly higher excellent or good outcomes than the control group did. In the meantime, the recurrence rates of undesirable symptoms after removing the fixing apparatus of the observation group were significantly lower than those of the control group (P<0.05) (Table-II).

## DISCUSSION

Distal clavicle fractures and shoulder joint dislocations, as common bone fracture types in clinical practice, are mainly triggered by violence.^5^ Distal clavicle fractures can be classified into three Neer types, while acromioclavicular joint dislocations can be classified into three Tossy types. In this study, Neer type II and Tossy type III patients were selected. Neer type II patients were complicated with rupture of coracoclavicular ligament attached to the proximal fracture. For Tossy type III patients, besides rupture of acromioclavicular ligament and coracoclavicular ligament, they also suffer from X-ray-disclosed complete distal displacement and piano key signs in some cases. It has previously been verified that Neer type II and Tossy type III patients could not be effectively treated by conservative protocols, which could be circumvented by open reduction internal fixation (ORIF). The results may be associated with the complete acromioclavicular joint displacement of Tossy type III patients that cannot be recovered by conservative treatment, which may thus give rise to upper limb dysfunction.^6^^-^^8^

**Table-I:** JOA scores before and after surgery

***Group***	***Case No.***	***Before surgery***	***Postoperative 6th week***	***Postoperative 12th week***
Observation group	50	55.4±3.7	83.2±1.8	97.4±1.5
Control group	50	54.8±4.5	71.6±2.2	82.3±2.6
t		1.029	5.873	6.271
P		>0.05	<0.05	<0.05

**Table-II:** Evaluation on therapeutic effects after surgery [case (%)].

***Group***	***Case No.***	***Excellent***	***Good***	***Poor***	***Rate of excellent and good outcomes (%)***	***Recurrence rate (%)***
Observation group	50	44 (88.0)	6 (12.0)	0	100%	0
Control group	50	17 (34.0)	13 (26.0)	20 (8.0)	60%	12.0%
χ^2^					25.000	6.383
P					<0.05	<0.05

On the contrary, ORIF can prevent hindered joint mobility and joint stiffness by facilitating ligament repair and early movement of joint. Hence, it is crucial to recover the functions of injured limbs by ORIF as early as possible. ORIF is commonly conducted by using steel wires, Kirschner wires and screws that easily lead to joint stiffness and pain by injuring joint and by evidently restricting the mobility of shoulder joint.^9^ For instance, Kirschner wires, which are prone to withdrawing in human body due to low firmness, are adverse to the early recovery of shoulder function. Recently, AO/ASIF clavicle hook plate has been applied in clinical practice to treat distal clavicle fractures and acromioclavicular joint dislocations. Designed based on the anatomical and biomechanics characteristics of the clavicle, this hook plate, which is made of titanium alloy to be adaptable to the "S" shape, is fixed on the clavicle, with its hook fixed under the acromion. The curved projection in the front of the distal plate end, which has a screw for fixation, fits perfectly to the enlarged region of the distal clavicle. By being fixed under the acromion, the plate is conducive to the fixation of screws by oppressing the distal clavicle.^10^^,^^11^

Moreover, AO/ASIF clavicle hook plate ensures stable fixation to prevent acromioclavicular joint dislocation or displacement. Without passing through the acromioclavicular joint, this plate hardly results in postoperative traumatic arthritis. In this study, the observation group was treated with AO/ASIF clavicle hook plates, and the control group was treated with Kirschner-wire tension bands. The JOA scores of the two groups were similar before surgery. Thereafter the two groups both had obviously increased JOA scores in the postoperative 6th and 12th weeks, and the score in the postoperative 12th week was higher. Significantly more patients in the observation group were evaluated as excellent or good outcomes after fixation than those in the control group. Meanwhile, after removal of the surgical apparatus, the recurrence rates of bone fracture and joint dislocation in the observation group were significantly lower than those of the control group. The results suggested that AO/ASIF clavicle hook plate promoted the recovery of shoulder joint function while minimizing the odds of recurrence.

Muramatsu et al.^12^ have reported similar results. Kirschner wires were inferior to AO/ASIF clavicle hook plate in the bone fracture healing and joint function recovery after ORIF. This plate functioned stably while barely affecting bone fracture healing upon acromioclavicular joint rotation. In summary, AO/ASIF clavicle hook plate can treat distal clavicle fractures and acromioclavicular joint dislocations safely and effectively, which is eligibly applicable in clinical practice owing to facile operation, minor trauma and early restoration of joint function.

***Conflicts of interest:*** The authors declared no conflicts of interest.
